# The green tea polyphenol epigallocatechin-3-gallate (EGCG) restores CDKL5-dependent synaptic defects *in vitro* and *in vivo*

**DOI:** 10.1016/j.nbd.2020.104791

**Published:** 2020-05

**Authors:** L. Trovò, C. Fuchs, R. De Rosa, I. Barbiero, M. Tramarin, E. Ciani, L. Rusconi, C. Kilstrup-Nielsen

**Affiliations:** a Center of Neuroscience, Dept. Biotechnology and Life Sciences (DBSV), University of Insubria, Varese, Italy; b Dept. Biomedical and NeuroMotor Sciences, University of Bologna, Bologna, Italy

**Keywords:** CDKL5, Epigallatocathechin-3-gallate, DYRK1A, Synaptic defects, AMPA-R, α-amino-3-hydroxy-5-methylisozasole-4-propionic acid receptor, CDD, CDKL5 deficiency disorder, CDKL5, cyclin-dependen kinase-like 5, DIV, days *in vitro*, DS, Down syndrome, DYRK1A, dual-specificity tyrosine-phosphorylation-regulated kinase, EGCG, eipgallatocathechin-3-gallate, GFP, green fluorescent protein, KO, knockout, PND, postnatal day, PSD95, post-synaptic density protein 95, WB, western blot, WT, wild-type

## Abstract

CDKL5 deficiency disorder (CDD) is a rare X-linked neurodevelopmental disorder that is characterised by early-onset seizures, intellectual disability, gross motor impairment, and autistic-like features. CDD is caused by mutations in the cyclin-dependent kinase-like 5 (*CDKL5*) gene that encodes a serine/threonine kinase with a predominant expression in the brain. Loss of CDKL5 causes neurodevelopmental alterations *in vitro* and *in vivo,* including defective dendritic arborisation and spine maturation, which most likely underlie the cognitive defects and autistic features present in humans and mice.

Here, we show that treatment with epigallatocathechin-3-gallate (EGCG), the major polyphenol of green tea, can restore defects in dendritic and synaptic development of primary *Cdkl5* knockout (KO) neurons. Furthermore, defective synaptic maturation in the hippocampi and cortices of adult *Cdkl5-*KO mice can be rescued through the intraperitoneal administration of EGCG, which is however not sufficient to normalise behavioural CDKL5-dependent deficits. EGCG is a pleiotropic compound with numerous cellular targets, including the dual-specificity tyrosine-phosphorylation-regulated kinase 1A (DYRK1A) that is selectively inhibited by EGCG. DYRK1A controls dendritic development and spine formation and its deregulation has been implicated in neurodevelopmental and degenerative diseases. Treatment with another DYRK1A inhibitor, harmine, was capable of correcting neuronal CDKL5-dependent defects; moreover, DYRK1A levels were upregulated in primary *Cdkl5-*KO neurons in concomitance with increased phosphorylation of Tau, a well-accepted DYRK1A substrate. Altogether, our results indicate that DYRK1A deregulation may contribute, at least in part, to the neurodevelopmental alterations caused by CDKL5 deficiency.

## Highlights

• Epigallocatechin-3-gallate (EGCG) restores CDKL5-dependent synaptic defects *in vitro;*
• Epigallocatechin-3-gallate (EGCG) restores CDKL5-dependent synaptic defects *in vitro;*
• DYRK1A levels and activity is increased in CDKL5 deficient neurons;

## 1 Introduction

CDKL5 deficiency disorder (CDD; OMIM #300672) is a severe neurodevelopmental disease caused by mutations in the cyclin-dependent kinase-like 5 (*CDKL5*) gene that is characterised by the early onset of drug resistant seizures, strong hypotonia, severe developmental delay, gross motor impairment, prominent autistic features, and some Rett syndrome-like traits (Fehr et al., 2013). Currently, no cure is available for CDD patients and there is an urgent need to develop new therapies capable of improving their quality of life.

Mutations in the X-linked *CDKL5* gene, which encodes a serine-threonine kinase, consist of missense, nonsense, or frame-shift mutations (Hector et al., 2017); a few cases with genomic duplications including *CDKL5* have been reported to have a clinical outcome that partially overlaps that of CDD (Szafranski et al., 2015). The incidence of CDD is currently estimated at 1:40.000 new-borns (Olson et al., 2019). The vast majority of patients are heterozygous females but more than 10% of CDD patients are hemizygous males (Olson et al., 2019). Missense mutations in CDKL5 fall almost exclusively within the catalytic domain and have so far been found to be loss-of-function mutations that reduce or abolish the catalytic activity (Bertani et al., 2006). Conversely, mutations causing the premature termination of the C-terminus lead to increased catalytic activity and protein stability and are constitutively confined to the nuclear compartment (Rusconi et al., 2008); these derivatives, if expressed, might thus act as gain- or loss-of-function mutants depending on the intracellular compartment.

CDKL5 is expressed in various tissues, but studies in mice have demonstrated that the protein is most abundant in brain where its expression gets significantly induced in the first two post-natal weeks and reaches highest levels in the cortex and hippocampus (Rusconi et al., 2008). At the subcellular level, CDKL5 is present in both the cytoplasm and the nucleus and the shuttling between these compartments is finely regulated by neuronal activity (Rusconi et al., 2011).

The role of CDKL5 for proper brain development and functioning is still not fully understood, but the combination of *in vitro* molecular studies on cultured primary neurons with *in vivo* phenotyping of the generated CDD mouse models has begun to shed light on the physiological functions of CDKL5 and on the etiology of CDD (Zhu and Xiong, 2019). In particular, *Cdkl5*-KO mice display several behavioural abnormalities reminiscent of the human symptomatology, including severe impairment of hippocampus-dependent learning and memory, visual, and respiratory deficits, motor impairment, and autistic-like features (Amendola et al., 2014; Jhang et al., 2017; Wang et al., 2012). Moreover, *Cdkl5*-KO mice show abnormal event related potentials, suggesting a pivotal role of CDKL5 for proper synaptic functioning and neuronal network formation (Wang et al., 2012). Behavioural deficits are accompanied by neurodevelopmental alterations, including aberrant neuronal precursor proliferation and survival as well as impaired axonal specification and elongation and defective dendritic arborisation (Fuchs et al., 2019, 2014; Nawaz et al., 2016). Moreover, loss of CDKL5 affects spine maturation and the expression and membrane exposure of the GluA2 subunit of glutamate α-amino-3-hydroxy-5-methylisozasole-4-propionic acid receptors (AMPA-R) both *in vivo* and *in vitro* (Fuchs et al., 2014; Ren et al., 2019; Tramarin et al., 2018; Yennawar et al., 2019).

From a molecular point of view, multiple signalling pathway components, including protein kinases such as AKT, GSK3β, AMPK, and PKA, and ERK, were found to be deregulated in the absence of CDKL5, suggesting that CDKL5 plays a role in regulating different neuronal signalling pathways (Fuchs et al., 2018, 2014; Wang et al., 2012).

Epigallatocathechin-3-gallate (EGCG) is the most abundant polyphenol found in green tea leaves (A. Islam, 2012). EGCG appears to have many actions on the brain, including its function as a powerful antioxidant, preventing oxidative damage in healthy cells. It also affects a wide array of pro-survival/differentiation signal transduction pathways, including ERK, PI3K/AKT, and DYRK1A (Shankar, 2007). Therefore, over the past few years it has garnered significant scientific interest as a therapeutic option for several neurological disorders (Granja et al., 2017; Guéroux et al., 2017). Treatment with EGCG is currently under testing in more than 90 clinical trials, including Fragile X and Down syndrome (DS) (https://clinicaltrials.gov); importantly, EGCG has been reported to be safe and effective in improving cognitive impairment in DS patients and its action has been postulated to occur through the inhibition of DYRK1A (de la Torre et al., 2016). Considering the many positive actions of EGCG on the brain, we deemed it intriguing to evaluate whether treatment with EGCG might have a positive impact in the context of CDD. Here we show that treatment with EGCG efficiently restores defects in dendritic and synaptic development of *Cdkl5*-KO hippocampal neurons. Even if *in vivo* treatment with EGCG has no effect on CDKL5-related behavioural deficits it rescues synaptic alterations. In fact, synaptic maturation is restored as well as the expression of post-synaptic density protein 95 (PSD95) and GluA2, two proteins involved in proper spine formation and function, in *Cdkl5-*KO mice (Fuchs et al., 2014; Ren et al., 2019; Tramarin et al., 2018; Yennawar et al., 2019).

## 2 Materials and methods

### 2.1 Antibodies and reagents

The following antibodies were used: anti-CDKL5 (Santa Cruz Biotechnology, sc-376314), anti-DYRK1A (Santa Cruz Biotechnology, sc-100376), anti-GAPDH (Sigma Aldrich, G9545), anti-GFP (Millipore AB16901), anti-MAP2 (Abcam mouse, ab11267; Abcam rabbit, ab32454), anti-NR2A (Merck Millipore, 07-632), anti-NR2B (Novus Biologicals, NB300-106), anti-PSD95 (Thermo Fischer Scientific, MA1045), anti-phospho-Tau-T^212^ (Invitrogen, 44-740G), anti-Tau (Santa Cruz Biotechnology, sc-322274).

HRP-conjugated goat anti-mouse or anti-rabbit secondary antibodies for immunoblotting and secondary Alexa Fluor anti-rabbit and anti-mouse antibodies for immunofluorescence were purchased from Thermo Fischer Scientific.

### 2.2 Primary neuronal cultures

Primary hippocampal cultures were prepared from mice of the *Cdkl5-KO* strain (Amendola et al., 2014) kept on a CD1 background. WT and *Cdkl5-*KO embryos were obtained crossing heterozygous females with WT males. Protocols and use of animals were approved by the Animal Ethics Committee of the University of Insubria and in accordance with the guidelines released by the Italian Ministry of Health (D.L. 2014/26) and the European Community directives regulating animal research (2010/63/EU). Brains of mouse embryos were collected at 18 days (E18), considering the day of vaginal plug as E0. The mice were sacrificed through cervical dislocation, then the embryos were recovered and the hippocampi were rapidly dissected. After washing in HBSS (Hank's Balanced Salt Solution - Gibco), the hippocampi were dissociated by 10 min incubation at 37 °C in 0.25% trypsin (Invitrogen). Cells were suspended in dissection medium [Dulbecco's Modified Eagle Medium, DMEM (Sigma-Aldrich), 10% horse serum (Euroclone), 2 mM l-Glutamine (Sigma), and 1 mM Sodium Pyruvate (Gibco)] to block the action of trypsin. Finally, cells were plated on poly-l-lysine hydrobromide coated dishes (0.1 mg/ml) or glass coverslips (1 mg/ml) at high density (30,000 cells/cm^2^) or at low density (3500 cells/cm^2^) in Neurobasal Medium [Neurobasal (NB) Medium supplemented with B27 (Gibco) and 2 mM l-Glutamine]. After 3 days *in vitro* (DIV3), cytosine-1-β-d-arabinofuranoside (Sigma-Aldrich) was added to cultured neurons at a final concentration of 2 μM to prevent astroglial proliferation. Primary hippocampal neurons were maintained in a humidified incubator with 5% of CO_2_ at 37 °C.

### 2.3 Pharmacological treatments

Primary hippocampal neurons were treated daily from DIV7 to DIV10 or from DIV14 to DIV17 with EGCG (epigallocatechin-3-gallate; Tocris) or harmine (Sigma Aldrich) dissolved in water. The final concentrations used were 0.1 μM, 0.5 μM, 1 μM, and 3 μM for EGCG and 0.05 μM, 0.1 μM, and 0.3 μM for harmine.

### 2.4 Neuronal transfection

Primary hippocampal neurons were transfected with the pCAGGS-IRES-GFP plasmid at DIV15, using the Lipofectamine 2000 reagent (Invitrogen). For each well of a 24-well plate, 0.2 μg of DNA were added to 100 μl of NB and 0.4 μl of Lipofectamine 2000 were added to 100 μl of NB. These solutions were allowed to stand for 5 min and were then mixed and incubated for 20 min at room temperature. The conditioned medium of neurons was removed and preserved at 37 °C. After incubation, the lipofectamine 2000/DNA mixture was added dropwise to neurons and then incubated for 45 min at 37 °C. Then the Lipofectamine 2000/DNA mixture was removed, cells were quickly rinsed in warm NB, and the preserved conditioned medium was added to the respective neurons. Neurons were fixed 2 days after transfection allowing the expression of GFP (green fluorescent protein).

### 2.5 Western blot

Cells were collected in sample buffer, supplemented with β-mercaptoethanol, after a rapid wash in PBS (Phosphate-Buffered Saline – Sigma-Aldrich), and then sonicated. Samples were separated by either 12 or 10% SDS-PAGE [Acrylamide/Bis-acrylamide solution 37.5:1 (Euro-Clone)], transferred to nitrocellulose membranes and blocked in 5% skimmed milk in Tris-Buffered Saline-Tween-20 (TBS-T): 20 mM Tris HCl pH 7.4, 150 mM NaCl, supplemented with 0,2% Tween-20 (Sigma-Aldrich). Blots were incubated with primary antibodies in blocking solution overnight at 4 °C, then washed in TBS-T and incubated with horseradish-conjugated secondary antibodies in blocking solution. After extensive washing, blots were developed with chemiluminescence-based detection system (StoS PSD, Genespin) coupled with G:BOX Chemi Imaging System (Syngene). Densitometric expression analyses of protein expression were performed using FiJi Software. Protein expression levels were normalised using GAPDH as internal standard. Phosphorylation was evaluated as the ratio between phosphoproteins and their total form.

### 2.6 Immunofluorescence

Primary hippocampal neurons plated on glass coverslips were fixed at DIV10 or DIV17 with fixing solution [4% paraformaldehyde, 4% sucrose and PBS] for 15 min and, after extensive washes, incubated with a permeabilising blocking solution [5% Horse Serum, 0,2%; Triton X100 in PBS] for more than 1 h at room temperature and then with primary antibodies overnight at 4 °C in blocking solution. After washing in PBS, neurons were incubated with secondary antibodies for 1 h at room temperature. Coverslips were mounted for microscopy using ProLong antifade reagent (Molecular Probes). Images were captured using 10×, 20×, and 60× objectives coupled with an Olympus BX51 Fluorescence microscope equipped with Retiga R1 (QImaging) CCD camera.

### 2.7 Image analysis and quantification

Acquired images of MAP2 staining, specifically identifying dendrites, were analysed thanks to the “Sholl Analysis” plugin of the FiJi software (htpp://Fiji.sc). Neurons were outlined to exclude adjacent cells or areas of non-specific immunoreactivity. For each cell, densitometric thresholds were set to remove background labelling and identify detailed cellular processes. Then, values of starting radius and radius step size were inserted: 30 μm (to exclude neuronal soma) and 10 μm, respectively. The number of processes intersecting each ring was provided by the software. The elaboration of these data allowed us generating a cumulative curve of the number of intersections of each cell, indicative of the branching complexity. By multiplying the number of intersections with the diameter of the ring, a cumulative curve of estimated total dendrite length for each cell was generated.

Spine morphology was analysed thanks to the software “Neuron Studio”. Firstly, 50 spines were loaded in the software as a reference: the software generated an algorithm that was used for the following analysis. Thanks to this setting, the program was able to categorise spines into four different classes: mushroom, thin, filopodia and stubby. Five to ten dendrites of around 30 μm per condition were analysed.

### 2.8 *In vivo* treatment and analyses

#### 2.8.1 Animal husbandry

The mice used in this work derive from the *Cdkl5*-KO strain in the C57BL/6N background developed in (Amendola et al., 2014) and backcrossed in C57BL/6J for three generations. Hemizygous *Cdkl5-*KO male mice were produced and genotyped as previously described (Amendola et al., 2014). Age-matched controls (wild-type (WT) littermates) were used for all experiments. The day of birth was designated as postnatal day (PND) zero and animals with 24 h of age were considered as 1-day-old animals (PND1). Mice were housed 3–5 per cage on a 12 h light/dark cycle in a temperature- (23 °C) and humidity-controlled environment with standard mouse chow and water *ad libitum*. The animals' health and comfort were controlled by the veterinary service. All research and animal care procedures were performed according to protocols approved by the Italian Ministry for Health and by the Bologna University Bioethical Committee. In this study, all efforts were made to minimize animal suffering and to keep the number of animals used to a minimum.

#### 2.8.2 Drug and treatment

EGCG (Tocris, Cat. No. 4524, >98% purity) was dissolved in water. *Cdkl5*-KO and WT mice were randomly assigned to the 4 experimental conditions and, starting from PND60, were treated with vehicle (water) or EGCG (25 mg/Kg) administered i.p. daily (between 9.00 and 10.00 am) for 30 days. Animals were behaviourally tested as indicated in Fig. 7 and sacrificed 24 h after the last injection. Brains were collected and processed as further described.

#### 2.8.3 Behavioural analyses

All behavioural studies were performed blinded to genotype and treatment. Mice were allowed to habituate to the testing room for at least 1 h before the test, and testing was performed at the same time of day (10.00 am – 4.00 pm). The sequence of the tests was arranged to minimize the effect of one test influencing the subsequent evaluation of the next test, and a minimum of 24 h was left between different tests.

#### 2.8.4 Marble burying

The marble burying test was performed by placing animals individually in a home-cage-like environment with 5 cm of unscented standard bedding material and 20 marbles (14.3 mm in diameter) arranged in a 4 × 5 matrix; the animals were left undisturbed for 30 min. The number of marbles that were at least two-thirds buried at the end of the trial was counted.

#### 2.8.5 Assessment of stereotypical jumps in the open field arena

To evaluate stereotypic movements, the number of stereotypical jumps (repetitive beam breaks <1 s) in the corners of an open field arena (50 × 50 cm, 20 min trial) were manually counted by a trained observer. The test chambers were cleaned with 70% ethanol between test subjects.

#### 2.8.6 Morris water maze

Hippocampal-dependent spatial learning and memory was assessed using the Morris water maze (MWM). Mice were trained to locate a hidden escape platform in a circular pool. The apparatus consisted of a circular water tank (1 m in diameter, 50 cm high) with a transparent round escape platform (10 cm^2^) placed in a fixed position. The tank was filled with tap water at a temperature of 22 °C up to 0.5 cm above the top of the platform, and the water was made opaque with milk. In the experimental room, intra-maze and extra-maze visual cues were placed to enable spatial orientation. Mouse behavior was automatically video-tracked (EthoVision 3.1; Noldus Information Technology B.V.). During training, each mouse was subjected to either 1 swimming session of 4 trials (day 1) or 2 sessions of 4 trials per day (days 2–5), with an intersession interval of 1 h (acquisition phase). Mice were allowed to search for the platform for up to 60 s. If a mouse did not find the platform, it was gently guided to it and allowed to remain there for 15 s. During the intertrial interval (15 s), mice were placed in an empty cage. The latency to find the hidden platform was used as a measure of learning. Twenty-four hours after the last acquisition trial, on day 6, the platform was removed and a probe test was run. Animals were allowed to search for the platform for up to 60 s. The latency of the first entrance into the former platform area was employed as measures of retention of acquired spatial preference.

#### 2.8.7 Hind-limb clasping

Animals were suspended by their tail for 2 min and hind-limb clasping was assessed independently by two operators from video recordings. A clasping event is defined by the retraction of limbs into the body and toward the midline. The time spent hind-limb clasping was expressed as percentage.

#### 2.8.8 Histological procedures

Animals were euthanized with isoflurane (2% in pure oxygen) and sacrificed through cervical dislocation. Brains were quickly removed and cut along the midline. The right hemisphere was processed for protein extraction and western blot (WB) analyses as described below. The left hemispheres were Golgi-stained using the FD Rapid GolgiStain TM Kit (FD NeuroTechnologies). Briefly, hemispheres were immersed in the impregnation solution containing mercuric chloride, potassium dichromate, and potassium chromate and stored at room temperature in darkness for 2–3 weeks. Hemispheres were cut with a microtome into 100 μm thick coronal sections that were then directly mounted onto gelatin-coated slides and were air dried at room temperature in the dark for an additional 2–3 days. After drying, sections were rinsed with distilled water and subsequently stained in the developing solution of the kit. All steps of sectioning, imaging, and data analysis were conducted blindly to genotype and treatment.

#### 2.8.9 Imaging and data analyses

A light microscope (LEITZ, Leica Microsystems) equipped with a motorized stage and focus control system and a color digital camera (CoolSNAP-Pro; Media Cybernetics) were used for neuronal tracing and to take bright-field images. Measurements were carried out using Image Pro Plus software (Media Cybernetics). A series of sections across the whole rostrocaudal extent of the hippocampal dentate gyrus were used for reconstruction of Golgi-stained neurons. Golgi-stained neurons (8–12 per animal) were sampled from the outer part of the granule cell layer and traced with a dedicated software custom-designed for dendritic reconstruction (Immagini Computer, Milan, Italy), interfaced with Image Pro Plus (Media Cybernetics). The dendritic tree was traced live, at a final magnification of 500×, by focusing into the depth of the section. The operator starts with branches emerging from the cell soma and after having drawn the first parent branch goes on with all daughter branches of the next order in a centrifugal direction. At the end of tracing, the program reconstructs the total dendritic length and the number of branches. Spines of hippocampal granule neurons and pyramidal neurons of layer II/III of the primary motor (M1) and primary somatosensory cortex (S1) were counted using a 100× oil immersion objective lens. Dendritic spine density was measured by manually counting the number of dendritic spines on dendritic segments. In each mouse, 15 dendritic segments (segment length: 10–30 μm) were analysed and the linear spine density was calculated by dividing the total number of counted spines by the length of the sampled dendritic segment. Based on their morphology, dendritic spines can be divided into five different classes, which fall into two categories (immature spines: filopodium-like, thin- and stubby-shaped; mature spines: mushroom- and cup-shaped), which also reflect their state of maturation. The total number of spines was expressed per 10 μm and the number of spines belonging to each class was counted and expressed as a percentage.

### 2.9 Statistical analyses

Data are presented as means ±SEM and their statistical relevance was analysed with Prism software (GraphPad). Specific statistical tests for each set of experiments are reported in the respective figure legend. Briefly, the comparison of two groups (usually WT *vs.* KO) was performed by Student's *t*-test. In case of more than 2 groups, *i.e.*, for pharmacological treatments, data were analysed using ANOVA followed by Dunnett's or Tukey's test for multiple comparisons. The significance of variation in spine density/morphology and neuronal maturation *in vivo* was calculated using 2-WAY ANOVA followed by Fisher's LSD. Behavioural data were analysed with the Shapiro-Wilk test for normality. Datasets with normal distribution were analysed for significance using 2-WAY ANOVA followed by Fisher's LSD. Datasets with nonparametric distribution were analysed using the Kruskal-Wallis test followed by Dunn's multiple comparison test. In all tests, probability values of *p* < .05 were considered as statistically significant.

## 3 Results

### 3.1 Treatment with EGCG restores dendritic arborisation of *Cdkl5*-KO neurons

**Fig. 1:**
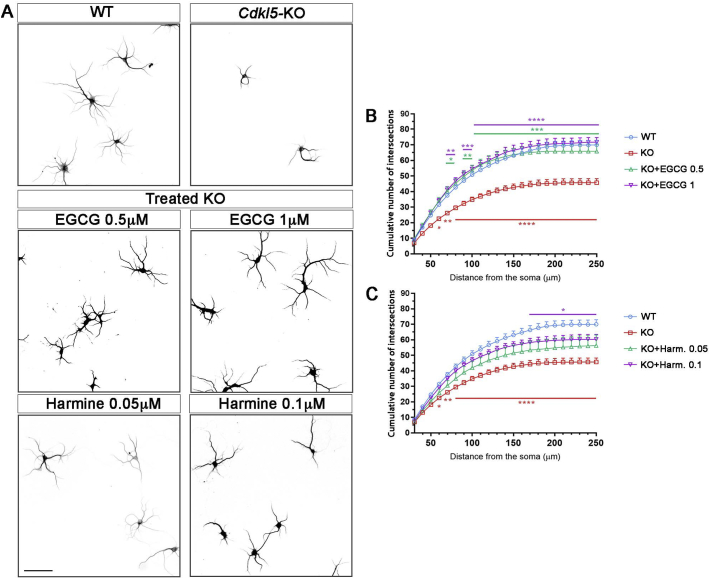
**Treatment with EGCG and har**min**e improves defective dendritic branching of *Cdkl5*-KO neurons *in vitro*. (A)** Representative images of untreated WT/*Cdkl5-*KO neurons and KO neurons treated with EGCG (0.5 μM and 1 μM) or harmine (0.05 μM and 0.1 μM) from DIV7-DIV10. Neurons were stained against MAP2 to identify dendrites. Images were inverted for clarity. **(B,C)** Quantification of dendritic branching represented as cumulative number of intersections in untreated WT/*Cdkl5-*KO neurons or KO neurons treated with EGCG (A) or harmine (C). 15 branches/mouse, *n* = 5–6 mice/group from at least 3 different preparations. Statistical analysis: 2-WAY ANOVA followed by Dunnett's post-hoc test (*p < .05, ** *p* < .01, ***p < .001, ****p < .0001). Scale bar 100 μm.

**Fig. S1:**
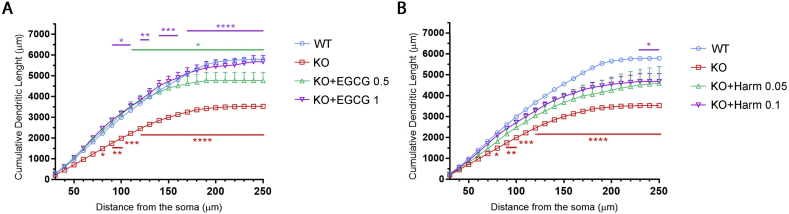
**Treatment with EGCG and harmine improves dendritic length of *Cdkl5*-null neurons *in vitro*. (A,B)** Quantification of cumulative dendritic length in untreated WT/*Cdkl5-*KO neurons or KO neurons treated with 0.5 μM and 1 μM EGCG (A) or 0.05 μM and 0.1 μM harmine (B). 15 branches/mouse, *n* = 5–6 mice/group from at least 3 different preparations. Statistical analysis: 2-WAY ANOVA followed by Dunnett's post-hoc test (*p < .05, **p < .01, ***p < .001, ****p < .0001).

To evaluate the effect of EGCG on neuronal maturation, we exploited primary cultures of hippocampal neurons that represent a well-accepted *in vitro* neuronal experimental system. Assessment of neurite length and complexity through Sholl analysis revealed in accordance with previous data (Trazzi et al., 2016) that *Cdkl5-*KO hippocampal neurons at DIV10 were less differentiated compared to WT neurons thus reproducing the defective arborisation of hippocampal granule neurons described *in vivo* (Amendola et al., 2014) (Fig. 1). In fact, *Cdkl5*-KO hippocampal neurons showed a significant reduction in the cumulative number of intersections (Fig. 1A,B; WT 70 ± 2.90, KO 45.7 ± 2.58) and in the cumulative dendritic length (WT 5788.6 ± 9.22 μm, KO 3526.4 ± 274.01 μm, Suppl. Fig. S1A).

Primary hippocampal cultures were treated daily from DIV7 to DIV10 with increasing concentrations of EGCG (0.1 μM, 0.5 μM, and 1 μM). While 0.1 μM EGCG was not effective in recovering dendrite branching in *Cdkl5-*KO neurons, treatment with EGCG at 0.5 μM and 1 μM concentrations restored the number of intersections as well as the total dendritic length in *Cdkl5-*KO neurons with respect to WTs (Fig. 1A,B and Suppl. Fig. S1A; cumulative number of intersections: WT 70 ± 2.90, KO 45.7 ± 2.58, KO + EGCG 0.1 μM 55.3 ± 11.44, KO + EGCG 0.5 μM 65.9 ± 5.55, KO + EGCG 1 μM 71.7 ± 4.24. Cumulative dendritic length: WT 5788.6 ± 9.22 μm, KO 3526.4 ± 274.01 μm, KO + EGCG 0.1 μM 4361.7 ± 1155,68 μm, KO + EGCG 0.5 μM 4765.1 ± 556.15 μm, KO + EGCG 1 μM 5676 ± 412.00 μm).

EGCG is a pleiotropic compound with a plethora of biological effects (Shankar, 2007). Among others, its inhibitory properties acting on the kinase DYRK1A have been extensively studied and described (IC_50_ = 330 nm) (Nguyen et al., 2017). In order to address the possible involvement of DYRK1A in the effect of EGCG on *Cdkl5-*KO neurons, we selected another potent and selective inhibitor of this kinase, harmine (IC_50_ = 80 nM) (Nguyen et al., 2017). In line with the above treatment schedule, we treated WT and *Cdkl5*-KO neurons daily from DIV7 to DIV10 with increasing concentrations of harmine (0.05 μM and 0.1 μM) and analysed the complexity of the dendritic arbor. Similarly to EGCG, also the treatment with harmine improved dendritic development in *Cdkl5*-KO neurons (Fig. 1A,C) reaching statistical significance with 0.1 μM harmine (Fig. 1C; cumulative number of intersections: KO 45.7 ± 2.58, KO + Harm 0.05 μM 56.3 ± 9.58, KO + Harm 0.1 μM 60.3 ± 4.08. Cumulative dendritic length: KO 3526.4 ± 274.01, KO + Harm 0.054592.7 ± 1153.85, KO + Harm 0.1 μM 4676.1 ± 535.34; Suppl. Fig. S1B).

### 3.2 Treatment with EGCG ameliorates defective spine maturation and number of PSD95^+^ puncta in *Cdkl5*-KO neurons

**Fig. 2:**
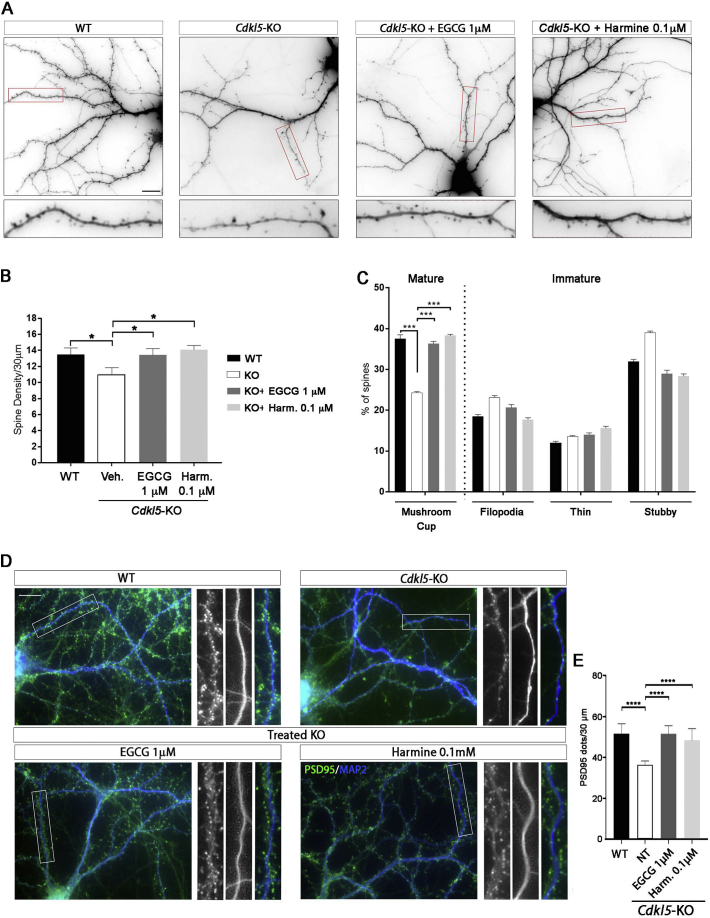
**Effect of EGCG and harmine on defective spine maturation of *Cdkl5*-KO neurons *in vitro*. (A)** Representative images of untreated WT/*Cdkl5-*KO neurons or KO neurons treated with 1 μM EGCG or 0.1 μM harmine from DIV14-DIV17. The transfection with GFP at DIV15 allowed the visualization of spines. Scale bar 10 μm. **(B)** Quantification of the number of spine protrusions in 30 μm-long dendritic segments of WT/*Cdkl5-*KO neurons treated as indicated. Statistical analysis: 1-WAY ANOVA followed by Dunnett's post-hoc test (*p < .05). **(C)** Morphological classification of spines in WT/*Cdkl5-*KO treated neurons, represented as percentage of total number of spines. Statistical analysis: 2-WAY ANOVA followed by Dunnett's post-hoc test (***p < .001). 5 branches/mouse, *n* = 3 mice/group from 3 different preparations. Data are presented as mean ± SEM. **(D)** Representative images of untreated WT/*Cdkl5-*KO neurons or KO neurons treated as indicated and stained with PSD95 (green) and MAP2 (blue) to visualise excitatory spines and dendrites, respectively. **(E)** Quantification of the number of PSD95^+^ dots in 30 μm-long dendritic segments of WT/*Cdkl5-*KO neurons treated as indicated. 10 branches/mouse, n = 3–8 mice/group from at least 3 different preparations. Data are presented as mean ± SEM. Statistical analysis: 1-WAY ANOVA followed by Dunnett's post-hoc test (****p < .0001). Scale bar 10 μm. (For interpretation of the references to color in this figure legend, the reader is referred to the web version of this article.)

In hippocampal neurons, CDKL5 mainly localises to dendritic spines and various reports show that loss of CDKL5 impairs synaptic connections and spine structures (Della Sala et al., 2016; Tramarin et al., 2018; Trazzi et al., 2016). To visualise spines on the dendritic shaft, hippocampal neurons were transfected with a GFP-expressing vector at DIV15 and fixed at DIV17. As shown in Fig. 2, *Cdkl5*-KO neurons showed a reduced number of dendritic spines in comparison with WT neurons. Importantly, daily treatment with EGCG (1 μM) or harmine (0.1 μM) from DIV14 to DIV17 significantly restored spine density (WT 13.5 ± 0.81, KO 11.06 ± 0.78, KO + Harm 0.1 μM 14.1 ± 0.52, KO + EGCG 1 μM 13.44 ± 0.78; Fig. 2A,B).

Dendritic spines are heterogeneous in size and shape and can be classified as immature spines (filopodia, thin, and stubby) and mature spines (mushroom/cup-shaped). Images were analysed with the NeuronStudio software to classify the spines into four different groups depending on their morphology. As shown in the graph (Fig. 2C), *Cdkl5*-KO neurons have a decreased number of mature spines (mushroom/cup) compared to WT neurons and a concomitant increase in immature spines (filopodia, thin and stubby) (mushroom/cup: WT 37.50 ± 0.37, KO 24.29 ± 0.25; filopodia: WT 18.52 ± 0.39, KO 23.16 ± 0.41; thin: WT 12.04 ± 0.35, KO 13.56 ± 0.22; stubby: WT 31.94 ± 0.50, KO 38.98 ± 0.43). Both EGCG and harmine were effective in increasing the percentage of mushroom-shaped mature spines in *Cdkl5*-KO neurons. Accordingly, a global decrease in the percentage of immature spines can be observed, with filopodial and stubby spines being mostly influenced (mushroom/cup: WT 37.50 ± 0.37, KO 24.29 ± 0.25, KO + Harm 0.1 μM 38.29 ± 0.30, KO + EGCG 1 μM 36.36 ± 0.53; filopodia: WT 18.52 ± 0.39, KO 23.16 ± 0.41, KO + Harm 0.1 μM 17.73 ± 0.42, KO + EGCG 1 μM 20.66 ± 0.74; thin: WT 12.04 ± 0.35, KO 13.56 ± 0.22, KO + Harm 0.1 μM 15.60 ± 0.49, KO + EGCG 1 μM 14.05 ± 0.39; stubby: WT 31.94 ± 0.50, KO 38.98 ± 0.43, KO + Harm 0.1 μM 28.37 ± 0.51, KO + EGCG 1 μM 28.92 ± 0.89; Fig. 2C).

PSD95 is a well-recognised marker of mature excitatory glutamatergic spines (Chen et al., 2011). As previously described, the number of PSD95^+^ puncta is reduced *in vivo* in brains of *Cdkl5-*KO mice, thus indicating an impairment in synaptic connectivity (Della Sala et al., 2016; Pizzo et al., 2016). To evaluate whether this defect can be reproduced in primary cultures of hippocampal *Cdkl5*-KO neurons and whether it can be reverted by treatment with EGCG we stained untreated and EGCG- and harmine-treated high-density cultures of *Cdkl5-*WT/KO neurons with antibodies against PSD95 and MAP2 to identify excitatory spines and dendritic shafts, respectively. For each image, a section of 30 μm was analysed with the FiJi software counting all PSD95^+^ dots. We observed a clear reduction in the number of countable PSD95^+^ dots in *Cdkl5*-KO neurons compared to the WT controls indicating that the absence of CDKL5 causes a significant reduction in the number of excitatory synaptic contacts also *in vitro* (Fig. 2D,E; WT 51.58 ± 2.21, KO 36.34 ± 0.68). Treatment with EGCG and harmine restored the number of PSD95^+^ dots in a strong and significant way (WT 51.58 ± 2.21, KO 36.34 ± 0.68, KO + Harm 0.1 μM 48.3 ± 2.36, KO + EGCG 0.5 μM 44.45 ± 3.12, KO + EGCG 1 μM 51.52 ± 1.52).

### 3.3 Loss of CDKL5 affects the DYRK1A pathway *in vitro*

**Fig. 3:**
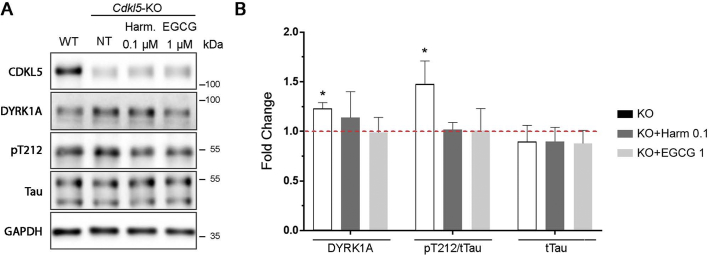
**The absence of CDKL5 alters the DYRK1A pathway. (A)** Representative WB showing the expression level of CDKL5, DYRK1A, total Tau and Tau phosphorylated on T212 in WT/*Cdkl5-*KO neurons or KO neurons treated with 1 μM EGCG or 0.1 μM harmine from DIV7-DIV10. GAPDH was used as internal standard. **(B)** Quantification of DYRK1A expression, the ratio between phosphorylated and total Tau (pT212/tTau) and total Tau levels in *Cdkl5-*KO neurons treated as indicated. Data are represented as fold change (mean ± SEM) compared to the untreated WT neurons indicated by the dotted horisontal line. Statistical analysis: 1-WAY ANOVA followed by Dunnett's post-hoc test (*p < .05). n = 3–10 mice/group from at least 3 different preparations.

EGCG and harmine are structurally unrelated DYRK1A inhibitors (Ionescu et al., 2012). The positive effect of treatment with EGCG and harmine on *Cdkl5*-KO neurons suggests that the DYRK1A pathway may be up-regulated in the absence of CDKL5. To address this, we first analysed DYRK1A expression through WB analysis of WT and *Cdkl5*-KO neurons collected at DIV10. We detected a slight but significant increase of DYRK1A protein levels in *Cdkl5*-KO neurons compared to WT controls (WT 1 ± 0.07, KO 1.23 ± 0.06; Fig. 3A,B). In line with increased DYRK1A expression we found higher phosphorylation levels of Tau on threonine 212 (T212), a well-described substrate of DYRK1A (Ionescu et al., 2012). While the total levels of Tau (tTau) remained constant, we detected an increase in the ratio between phosphorylated (pT212) and tTau in *Cdkl5*-KO neurons (pT212/tTau: WT 1 ± 0.08, KO 1.48 ± 0.23. tTau: WT 1 ± 0.02, KO 0.9 ± 0.16; Fig. 3A,B). As expected, treatment with the DYRK1A inhibitors EGCG or harmine normalised the pT212/tTau ratio in *Cdkl5-*KO neurons (pT212/tTau: WT 1 ± 0.08, KO 1.48 ± 0.23, KO + Harm 0.1 μM 1.02 ± 0.07, KO + EGCG 1 μM 1.01 ± 0.22) without affecting either total Tau levels (Fig. 3A,B; tTau: WT 1 ± 0.02, KO 0.09 ± 0.16, KO + Harm 0.1 μM 0.90 ± 0.14, KO + EGCG 1 μM 0.88 ± 0.13) or DYRK1A expression (DYRK1A: WT 1 ± 0.07, KO 1.23 ± 0.06, KO + Harm 0.1 μM 1.14 ± 0.26, KO + EGCG 1 μM 0.99 ± 0.15).

### 3.4 Treatment with EGCG has no detrimental effect on WT neurons

DYRK1A is a paradigm of a dosage sensitive gene with both its gain and loss of function impacting neuronal functions (Duchon and Herault, 2016). Due to random X-inactivation, female heterozygous CDD patients are mosaics with two neuronal populations expressing either WT or mutated CDKL5. We therefore found it relevant to test the effect of EGCG also on WT neurons to exclude possible negative effects of DYRK1A inhibition.

**Fig. S2:**
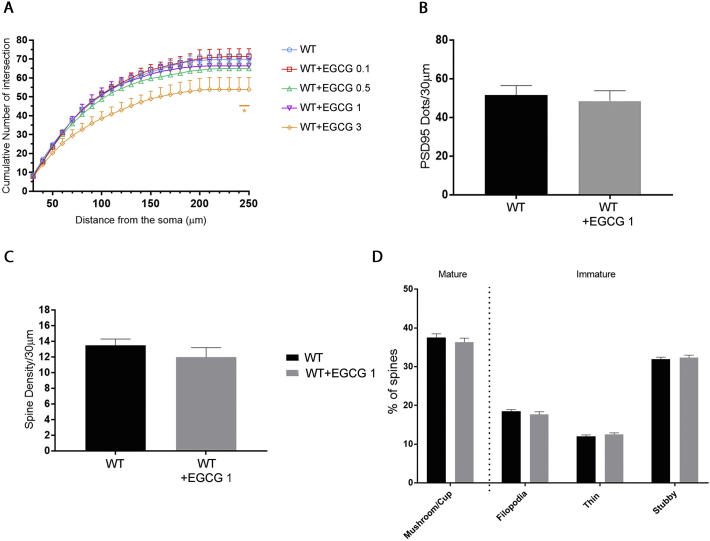
**Treatment with EGCG and harmine has no detrimental effect on *Cdkl5-*WT neurons. (A)** Quantification of the cumulative number of intersections in WT neurons treated with 0.1, 0.5, 1, or 3 μM of EGCG. 15 branches/mouse, n = 5–6 mice/group from at least 3 different preparations. Statistical analysis: 2-WAY ANOVA followed by Dunnett's post-hoc test. **(B)** Quantification of the number of spine protrusions in 30 μm-long dendritic segments of WT neurons treated with 1 μM EGCG or left untreated. 5 branches/mouse, *n* = 3 mice/group from 3 different preparations. Statistical analysis: Student's *t*-test. **(C)** Morphological classification of spines in WT neurons treated as in B, represented as percentage of total number of spines. 5 branches/mouse, n = 3 mice/group from 3 different preparations. Statistical analysis: 2-WAY ANOVA followed by Dunnett's post-hoc test. **(D)** Quantification of PSD95^+^ puncta of WT neurons treated with 1 μM EGCG or left untreated. 10 branches/mouse, n = 3–8 mice/group from at least 3 different preparations. Statistical analysis: Student's t-test. Data are presented as mean ± SEM.

WT neurons were treated from DIV7-10 with increasing concentrations of EGCG (0.1 μM, 0.5 μM, 1 μM, and 3 μM) and neuronal maturation, number of dendritic intersections, PSD95^+^ puncta, and spine morphology were evaluated. EGCG concentrations above 1 μM were found to impact negatively the dendritic development of WT neurons (Suppl. Fig. S2A) (cumulative number of intersections: WT 70 ± 2.90, WT + EGCG 0.1 μM 71.4 ± 4.07, WT + EGCG 0.5 μM 64.9 ± 4.26, WT + EGCG 1 μM 66.4 ± 4.54, WT + EGCG 3 μM 53.9 ± 6.30) while spine density and the overall morphological distribution of spines were not affected upon treatment with 1 μM EGCG from DIV14-DIV17 (Spine density: WT 13.5 ± 0.81, WT + EGCG 1 μM 12 ± 1.21) (Spine morphology: mushroom/cup: WT 37.50 ± 0.37, WT + EGCG 1 μM 36.37.5 ± 0.98; filopodia: WT 18.52 ± 0.39, WT + EGCG 1 μM 17.71 ± 0.63; thin: WT 12.04 ± 0.35, WT + EGCG 1 μM 12.5 ± 0.42; stubby: WT 31.94 ± 0.50, WT + EGCG 1 μM 32.29 ± 0.69; Suppl. Fig. S2C,D). Finally, we analysed the effect of EGCG on the number of PSD95^+^ dots in WT neurons: none of the tested concentrations were found to significantly affect the number of PSD95^+^ puncta in WT neurons (WT 51.58 ± 2.21; WT + EGCG 0.5 μM 46.46 ± 1.52; WT + EGCG 1 μM 48.44 ± 2.42; Suppl. Fig. S2B).

### 3.5 Treatment with EGCG restored spine density and maturation in brains of *Cdkl5*-KO mice

**Fig. 4:**
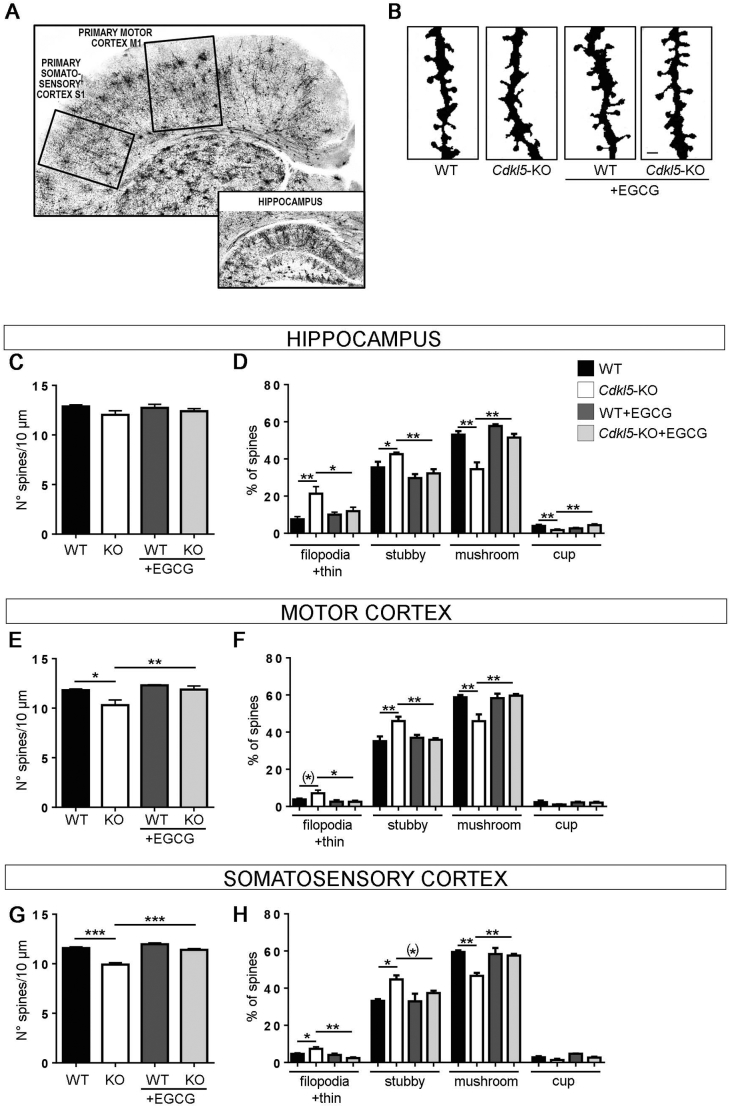
EGCG supplementation corrects CDKL5-dependent synaptic defects *in vivo*. **(A)** Representative images of Golgi-stained coronal sections containing the primary motor (M1) and primary somatosensory (S1) cortex (upper image) and the hippocampus (lower image). **(B)** Representative images of dendritic protrusions of granule neurons in WT/*Cdkl5*-KO male mice treated with EGCG (25 mg/Kg, dialy i.p.) or vehicle for 30 days (PND60–90). Scale bar 1 μM. **(C,E,G)** Dendritic spine density (number of spines per 10 μm) in hippocampal granule neurons (C), and pyramidal neurons of layer II/III of the primary motor (E) and primary somatosensory cortex (G) in vehicle-treated (granule neurons: WT *n* = 6, KO n = 6; pyramidal neurons WT n = 3, KO n = 3) and EGCG-treated (granule neurons: WT n = 3, KO *n* = 4; pyramidal neurons WT n = 3, KO n = 3) WT/*Cdkl5-*KO male mice. **(D,F,H)** Morphological classification of spines in mice as in C,E,G. Data are presented as mean ± SEM. Statistical analysis: 2-WAY ANOVA followed by Fisher's LSD ((*)*p* = .06, *p < .05, **p < .01, ***p < .001).

Given the capability of EGCG to robustly rescue the defects linked to the loss of CDKL5 *in vitro*, we decided to conduct a small pilot experiment to test whether a similar rescue could also be detected *in vivo*. WT and *Cdkl5*-KO male mice were randomly divided into four experimental groups (WT + Vehicle, WT + EGCG, KO + Vehicle, KO + EGCG; 5–9 mice/group) and treated with EGCG 25 mg/Kg, i.p., daily for 30 days, from PND60 to PND90 and the effects on neuroanatomical alterations, *i.e.* spine maturation and dendritic development, were evaluated at the end of the treatment. Although dendritic spine density in granule neurons was not different between vehicle-treated WT and *Cdkl5*-KO mice (Fig. 4 B,C), dendritic spines in the dentate gyrus of vehicle-treated *Cdkl5-*KO mice appear longer and thinner compared to WT (Fig. 4B) in accordance with previous reports (Fuchs et al., 2018, 2014; Trazzi et al., 2016). The quantification supported this observation, showing an increased percentage of immature spines (filopodial/thin, and stubby) with a concomitant decrease in mature spines (mushroom and cup) in *Cdkl5*-KO mice (Fig. 4 D). Similar to the results *in vitro*, the dendritic spines of EGCG-treated *Cdkl5*-KO animals presented a clear morphological amelioration resembling those of WT mice (Fig. 4B,D). A normalisation of the number of mature spines (mushroom and cup) with the concomitant reduction the immature spines (filopodia/thin, stubby) in EGCG-treated mice was evident upon spine classification and quantification (Fig. 4B,D), suggesting that EGCG also has positive effects on hippocampal spine maturation *in vivo*. To assess whether a similar EGCG-induced beneficial effect on spine maturation was present also in other brain structures, we further analysed spine density and spine maturation in pyramidal neurons of layer II/III of the primary motor (Fig. 4A,E,F) and the primary somatosensory cortex (Fig. 4A,G,H). In accordance with previous evidence (Della Sala et al., 2016; Fuchs et al., 2018), vehicle-treated *Cdkl5*-KO showed a decreased spine density in both analysed brain structures compared to their WT counterparts (Fig. 4E,G). Importantly, treatment with EGCG completely restored spine density in layer II/III apical dendrites of pyramidal neurons in the motor (Fig. 4E) and somatosensory cortex (Fig. 4G) of *Cdkl5*-KO mice. Furthermore, analysing the distribution of spines in the distinct morphology classes, we found a lower proportion of mature spines (mushroom and cup) and an increased fraction of filopodia-like, thin and stubby-shaped spines in cortices of vehicle-treated *Cdkl5*-KO mice (Fig. 4F,H). Treatment with EGCG completely restored the balance between immature and mature spines in both analysed cortical structures (Fig. 4F,H). Interestingly, spine density and morphology were not affected by EGCG-treatment in WT mice.

**Fig. 5:**
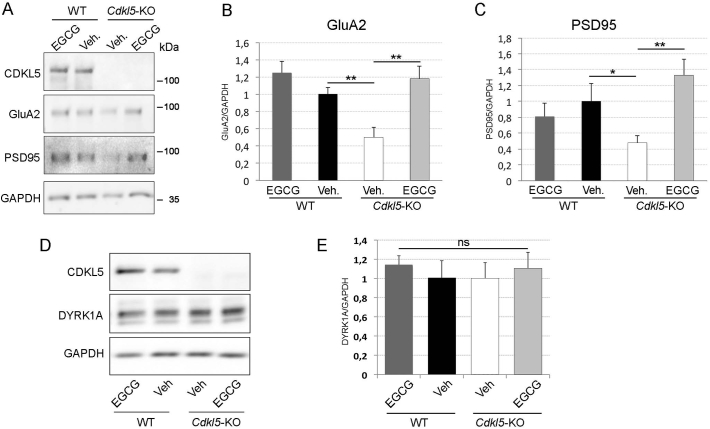
EGCG supplementation corrects CDKL5-dependent synaptic defects *in vivo*. **(A)** Representative WB of hippocampal total lysates from mice treated as described in Fig. 4. **(B,C)** Quantification of GluA2 (B) and PSD95 (C) expression in hippocampi of WT/*Cdkl5-*KO mice upon normalisation with the internal standard GAPDH. n = 5 mice/group. Data are presented as mean ± SEM. Statistical analysis: 2-WAY ANOVA followed by Fisher's LSD. *p < .05, **p < .01, ***p < .001. **(D,E)** WB analysis (D) and relative quantification (E) of DYRK1A expression in hippocampal total lysates from PND90 mice treated as in A and normalised to GAPDH. n = 5 mice/group. Data are presented as mean ± SEM. Statistical analysis: 2-WAY ANOVA followed by Fisher's LSD.

**Fig. S3:**
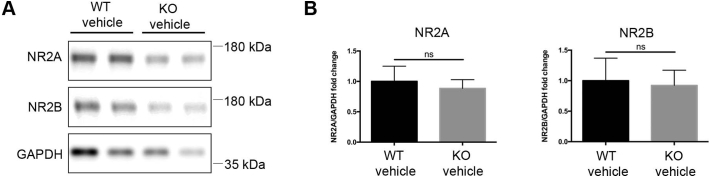
**Expression levels of NMDA-R subunits NR2A and NR2B are unaltered in hippocampal lysates of *Cdkl5-*KO mice. (*A*)** Representative WB showing expression of NMDA-R subunits NR2A and NR2B in hippocampi of *Cdkl5-*WT and KO mice. **(*B*)** Quantification of NR2A and NR2B levels upon normalisation with the internal standard GAPDH. Data are expressed as fold change compared to *Cdkl5-*WT samples (mean ± SEM). Statistical analysis: Unpaired *t*-test. n = 5 mice/group. Of note, NMDA-R expression was previously found increased in post-synaptic density fractions of *Cdkl5-*KO hippocampi whereas levels were unaltered in the post-nuclear fraction (Okuda et al., 2017), which is the most comparable to the total lysates of our study.

As previously observed *in vitro* in CDKL5 silenced neurons (Tramarin et al., 2018) and *in vivo* in hippocampi and cortices of *Cdkl5-*KO mice (Ren et al., 2019; Yennawar et al., 2019) we found reduced expression of the GluA2 AMPA-R subunit in the hippocampus of *Cdkl5-*KO mice in comparison with WT mice (Fig. 5A,B) whereas the loss of CDKL5 did not alter the expression of NMDA-R subunits NR2A and NR2B (Suppl. Fig. S3). Notably, GluA2 levels were rescued in EGCG-treated *Cdkl5-*KO mice. Similar to the rescue of the structural defect, we found that EGCG treatment also restored the expression of PSD95 to WT levels (Fig. 5A,C). GluA2 and PSD95 levels in EGCG-treated WT mice were not significantly different compared to those in vehicle-treated WT animals (Fig. 5A–C).

Since the DYRK1A pathway appeared to be hyperactive in the absence of CDKL5 *in vitro*, we decided to test if this could be reproduced also *in vivo*. Hippocampi from vehicle/EGCG-treated WT/*Cdkl5-*KO mice were analysed for the expression of DYRK1A by WB. Contrary to what we observed *in vitro,* DYRK1A levels were not influenced either by the genotype or by treatment (Fig. 5D,E).

### 3.6 Treatment with EGCG has no beneficial effects on dendritic development and behaviour in *Cdkl5*-KO mice

**Fig. 6:**
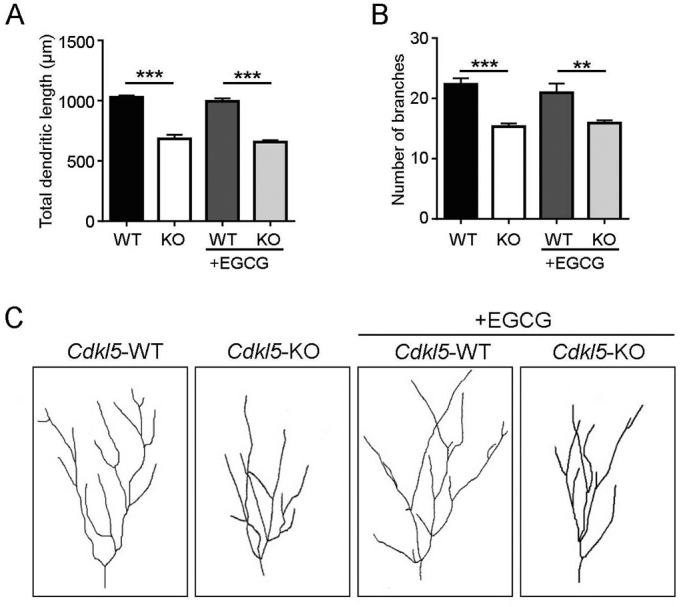
**EGCG supplementation has no effect on dendritic development in *Cdkl5*-KO mice. (A,B)** Mean total dendritic length (A) and mean number of dendritic branches (B) of Golgi-stained granule neurons of vehicle-treated (WT n = 4, KO n = 3) and EGCG-treated (WT n = 3, KO n = 3) WT/*Cdkl5-*KO male mice. **(C)** Examples of the dendritic tree of Golgi-stained granule neurons of 1 animal from each experimental group. Data are presented as mean ± SEM. Statistical analysis: 2-WAY ANOVA followed by Fisher's LSD (**p < .01, ***p < .001).

In order to establish whether treatment with EGCG, in addition to spine maturation, was able to rescue dendritic development *in vivo,* we examined the dendritic pattern of Golgi-stained granule neurons in vehicle- and EGCG-treated mice. In line with previous evidence (Fuchs et al., 2018; Fuchs et al., 2015; Ren et al., 2019; Trazzi et al., 2018), granule neurons from vehicle-treated *Cdkl*5-KO mice had a significantly reduced total dendritic length (Fig. 6 A,C) and fewer branches (Fig. 6 B,C) compared to their WT counterparts. Differentially, from what was observed *in vitro,* treatment with EGCG did not improve these parameters in *Cdkl5*-KO mice (Fig. 6A–C).

**Fig. 7:**
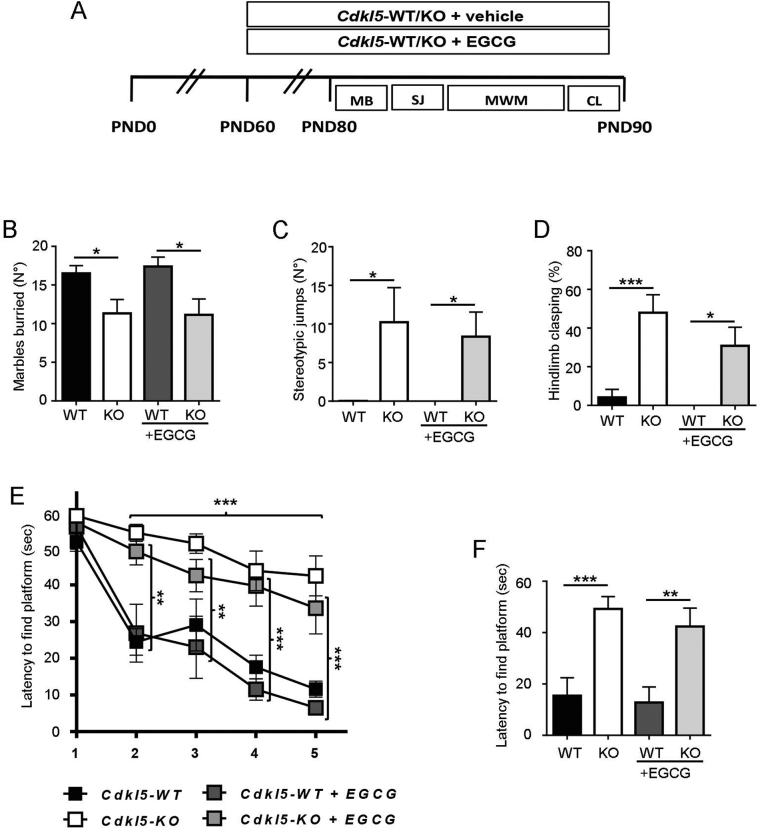
**EGCG supplementation has no effect on behavioural abnormalities in *Cdkl5*-KO mice. (A)** Experimental protocol. Starting from PND 60, WT/*Cdkl5*-KO male mice were treated either with vehicle or EGCG (25 mg/Kg, daily i.p.) for 30 days (PND60 to 90). From PND80, after 20 days of treatment, mice were subjected to the following behavioural tests as indicated: MB, Marble burying; SJ, Stereotypical jumps, MWM, Morris water maze, CL, Hind-limb clasping. **(B)** Number of marbles buried in vehicle-treated (WT *n* = 8, KO *n* = 9) and EGCG-treated (WT n = 5, KO *n* = 7) WT/*Cdkl5-*KO male mice. **(C)** Number of stereotypical jumps in the corners of the open field arena during a 20 min trial in mice as in B. **(D)** Percentage of time spent hind-limb clasping during a 2 min interval in mice as in B. **(E)** Spatial learning (5-day learning period) assessed using the Morris water maze in mice as in B. **(F)** Spatial memory on day 6 (probe test) was assessed by evaluating the latency to enter the former platform zone. Data are presented as mean ± SEM. Statistical analysis: (C) Dunn's test after Kruskal-Wallis, (B, D-F) 2-WAY ANOVA followed by Fisher's LSD (*p < .05, **p < .01, ***p < .001).

To test whether EGCG-induced restoration of spine density and maturation in *Cdkl5*-KO mice is sufficient to lead to an improvement in behavioural abnormalities, vehicle-treated and EGCG-treated mice were behaviourally tested as indicated in Fig. 7A. We assessed the effects of EGCG-treatment on autistic-like phenotypes (*i.e.* marble burying (Fig. 7B), stereotypical jumps (Fig. 7C), hind-limb clasping (Fig. 7D), and hippocampus-dependent learning and memory (Morris water maze; Fig. 7E,F). In accordance with previous reports (Fuchs et al., 2015, 2018; Trazzi et al., 2016) vehicle-treated *Cdkl5*-KO mice were severely impaired in all these behavioural tests. Treatment with EGCG had no beneficial effects on behavioural abnormalities in *Cdkl5*-KO mice (Fig. 7B–F).

## 4 Discussion

CDD is a rare disorder caused by mutations in the X-linked *CDKL5* gene that encodes for the homonymous protein. To date, no cure exists for CDD and the existing therapies are purely symptomatic. To facilitate the identification of new relevant disease modifying molecules, we have exploited an *in vitro* platform of primary WT/*Cdkl5-*KO hippocampal neurons that reproduce defects already reported *in vivo* in the mouse model of the disease such as reduced dendritic branching and deranged dendritic spine maturation (Amendola et al., 2014; Chen et al., 2010; Della Sala et al., 2016; Pizzo et al., 2016; Ricciardi et al., 2012; Trazzi et al., 2016). While the *in vitro* platform is a simplified model compared to the *in vivo* model, it allows for a faster and reliable phenotypic screening to evaluate the efficacy of a drug without the confounding effect of the heterogeneous population of cells in the brain. We thus utilised this *in vitro* platform and tested the therapeutic potential of EGCG: this is a polyphenol extracted from green tea that is currently under clinical investigation. Indeed, more than 90 clinical trials are testing the therapeutic potential of EGCG for a number of diseases, such as cancer, heart disease, diabetes, obesity, and several neurological disorders including Fragile X and Down syndromes (https://clinicaltrials.gov) (de la Torre et al., 2016; Granja et al., 2017; Guéroux et al., 2017). To this regard, cognitive defects in young DS patients were improved by EGCG without any adverse effects (de la Torre et al., 2016). Our results demonstrated the efficacy of EGCG-treatment in reverting all the CDKL5-dependent defects *in vitro*: in fact, defective dendritic arborisation, in terms of cumulative number of intersections and dendritic length, as well as spine density and morphology and PSD95^+^ puncta were efficiently normalised to control levels. These data indicate that EGCG is capable of consistently improving the overall maturation state of *Cdkl5*-KO neurons *in vitro* by increasing the complexity of the dendritic arbor and implementing functional synaptic connectivity. It is worth of note that neurons were treated when synaptic and dendritic defects were already full-blown. The capacity of EGCG to revert these defects, rather than preventing their appearance, provides stronger support for the therapeutic potential of EGCG for CDD patients.

EGCG is a pleiotropic compound with countless biological properties and cellular targets, such as cell cycle proteins, transcription factors, protein kinases, and anti-apoptotic or apoptotic proteins (Shankar, 2007). Among these, EGCG presents the lowest IC_50_ for the kinase DYRK1A (IC_50_ = 330 nm) (Nguyen et al., 2017). DYRK1A is particularly interesting since its expression and activity have previously been reported to modulate dendritic development (Göckler et al., 2009). Interestingly, also spine density and morphology are regulated by DYRK1A. In particular, the DYRK1A-mediated phosphorylation of N-WASP, a protein involved in actin polymerisation and dynamics, impairs spine formation (Park et al., 2012).

To evaluate whether the observed effects of EGCG on *Cdkl5*-KO neurons can be ascribed, at least partially, to DYRK1A inhibition, we also tested the effect of harmine. In contrast to EGCG, harmine is an ATP-competitive inhibitor of DYRK1A and is structurally unrelated to EGCG (Ionescu et al., 2012). Besides DYRK1A, harmine has several off-targets, but DYRK1A is the only common target between the two compounds. Treatment with harmine was capable of correcting CDKL5-dependent phenotypes: indeed, dendritic branching as well as spine maturation was significantly ameliorated by harmine. Altogether, the positive effects exerted by both DYRK1A inhibitors strongly suggest that DYRK1A signalling may be up-regulated in the absence of CDKL5, thus contributing to the morphological deficits in *Cdkl5*-KO neurons. Interestingly, we could detect a slight but significant increase in DYRK1A expression in *Cdkl5*-KO neurons. Furthermore, the phosphorylation of Tau on T212, a well-accepted DYRK1A substrate that is widely used as readout of the catalytic activity of DYRK1A (Ionescu et al., 2012), was augmented in *Cdkl5-*KO neurons. It is relevant to mention that treatment with both harmine and EGCG caused a decrease in the phosphorylation state of Tau in line with an inhibition of DYRK1A in treated cells. Taken together, these results demonstrate that the absence of CDKL5 induces an up-regulation in the DYRK1A pathway and provide the rationale for the capacity of DYRK1A inhibitors to correct CDKL5-dependent neuronal defects.

The observed up-regulation of the DYRK1A pathway in *Cdkl5-*KO neurons is comparable to that observed in DS patients (Guimera et al., 1999). Moreover, pathological mutations in DYRK1A cause phenotypic outcomes (autism, seizures, cognitive impairment) and molecular defects (neural progenitor proliferation, microtubule dynamics, dendritic development, spine maturation) that greatly overlap with those linked to CDKL5 (Arbones et al., 2019). Importantly, DYRK1A is a dosage-sensitive gene: both its up- and down-regulation negatively impact neuronal morphology and development (Dang et al., 2018), and both gain- and loss-of-function pathological mutations of DYRK1A have been reported so far. Indeed, patients carrying loss-of-function mutations present some features similar to DS patients such as intellectual disabilities and recurrent dysmorphic features, while also microcephaly, visual impairment, abnormal MRI scans and seizures are frequent (Evers et al., 2017). Thus, since DYRK1A is a paradigm of a dosage-sensitive gene, its inhibition may have a therapeutic potential in CDKL5 deficient neurons while on the same time being detrimental for WT neurons. Considering that most CDD patients are heterozygous females, and hence mosaics with neurons expressing either WT or mutated CDKL5, we found it relevant to test also the impact of EGCG on WT neurons to exclude any detrimental effects.

Importantly, the concentrations that were effective on *Cdkl5-*KO neurons did not display any negative effects on WT neurons (harmine: 0.1 μM, EGCG: 0.5–1 μM) in terms of dendritic development and spine morphogenesis. Conversely, as expected, the excessive inhibition of DYRK1A (harmine: 0.3 μM, EGCG: 3 μM) induced a reduction of the dendritic branching, confirming the previously reported dose sensitivity of DYRK1A (Copf, 2015).

Given the convincing results obtained *in vitro* and the feasible translation of EGCG into clinical applications, we decided to evaluate if the beneficial effect of EGCG was reproduced also in a complex organism. In *Cdkl5-*KO mice that were already symptomatic, treatment with this polyphenol efficiently restored synaptic connectivity in terms of both spine morphology and number of functional glutamatergic contacts. These improvements were accompanied by a normalisation of the expression of PSD95 and of the AMPA-R subunit GluA2. Unexpectedly, the positive effects on synaptic connectivity seemed to be unrelated to DYRK1A: indeed, differently from the *in vitro* results, the expression levels of DYRK1A were similar in hippocampi of WT and *Cdkl5*-KO mice. Moreover, differentially from what observed *in vitro*, treatment with EGCG was not able to reshape the dendritic pattern of hippocampal granule neurons in *Cdkl5*-KO mice. This incongruity is probably due to the fact that the *in vitro* model represents a simplified system that reveal subtle molecular alterations that are lost in a complex and intricate *in vivo* brain network. Although our results show that pharmacotherapy with EGCC fully rescues spine maturation and synaptic connectivity in *Cdkl5-*KO mice, these effects do not lead to a behavioural improvement. Indeed, behavioural testing of the treated mice did not reveal an improvement on key deficits linked to CDKL5 deficiency. The apparent inefficiency of EGCG treatment in improving the behavioural phenotypes is somewhat unexpected considering its positive effect on synaptic CDKL5-dependent defects. However, since we did not observe any effect of EGCG treatment on dendritic complexity *in vivo* it is possible that the treatment protocol was not sufficient to restore a more complete spectrum of CDKL5 related neuroanatomic defects thus not leading to a normalisation of behavioural phenotypes. Our treatment protocol (25 mg/kg through daily i.p. injections for 30 days) was based on literature showing that higher doses may have hepatotoxic (Ramachandran et al., 2016) and pro-apoptotic (Singh et al., 2016) effects. Accordingly, we observed a negative effect of higher doses of EGCG (3 μM) on WT neurons *in vitro* and do therefore not consider beneficial treating mice with a higher dose of EGCG. However, we consider worth testing through future studies whether the combined action of EGCG treatment with other pro-cognitive drugs might lead to a synergistic effect on behavioural defects in *Cdkl5-*KO mice.

To conclude, we have collected convincing evidence that dietary supplementation of EGCG, in combination with the standard pharmacological intervention, may have a positive impact on CDKL5-related neurological defects, although additional studies will be required to further investigate its therapeutic potentiality. Moreover, we demonstrated that DYRK1A signalling is deranged *in vitro* in the absence of CDKL5, shedding light on novel molecular perspectives for this kinase. The positive effect of EGCG supplementation on synaptic connectivity *in vivo* without any perturbation of DYRK1A levels and the fact that, conversely to EGCG, harmine exerts only a partial amelioration of dendritic branching *in vitro* suggest that other unexplored molecular mechanisms beside DYRK1A may underlie the effect of EGCG. Further investigations are needed to unveil this issue.

## Funding

This work was supported by the Telethon Foundation (GGP15098 to EC and CKN), by a junior fellowship to LR from the Loulou Foundation and by the Italian Parents' Association Albero di Greta to CKN.

## Declaration of Competing Interest

None.

The following are the supplementary data related to this article.

Supplementary data to this article can be found online at https://doi.org/10.1016/j.nbd.2020.104791.
